# Clinical and radiographic evaluation of pulpectomy in primary teeth: a 18-months clinical randomized controlled trial

**DOI:** 10.1186/s13005-017-0145-1

**Published:** 2017-10-27

**Authors:** Xiaoxian Chen, Xinggang Liu, Jie Zhong

**Affiliations:** 1 Department of Pediatric Dentistry, First clinical Division, Peking University School and Hospital of Stomatology, Beijing, China; 2 Department of Prosthodontics, Beijing Stomatological Hospital&School of Stomatology, Capital Medical University, 4 Tian Tan Xi Li, Beijing, 100050 People’s Republic of China

**Keywords:** Pulpectomy, Primary teeth, Zinc Oxide-Eugenol, Iodoform, Calcium hydroxide

## Abstract

**Background:**

To avoid untoward changes when primary teeth are replaced by permanent teeth, resorption of the material used in primary teeth root canal filling should occur at the same rate as root resorption. The Aim of this study was to compare the success rates of a mixed primary root canal filling (MPRCF, ingredients: zinc oxide–eugenol [ZOE], iodoform, calcium hydroxide) to those of ZOE and Vitapex in pulpectomised primary molars.

**Methods:**

One hundred and sixty primary molars from 155 children (average age 5.88 ± 1.27 years) underwent two-visit pulpectomy using one of the three materials. The clinical and radiographic findings at 6, 12 and 18 months were assessed.

**Results:**

At 6 and 12 months, the MPRCF and ZOE success rates were 100%. The Vitapex group showed clinical success rate and radiographic success rate of 100 and 94.5% at 6 months, and 80.4 and 60.7% at 12 months. The 18-month clinical success rates of the MPRCF, ZOE and Vitapex were 96.2, 92.2 and 71.4% and radiographic success rates were 92.5, 88.2 and 53.6%, respectively. There was a statistically significant difference in the success rates between MPRCF and Vitapex and no significant differences between MPRCF and ZOE. More MPRCF were resorbed at same rate with roots than ZOE and Vitapex. Early resorption of root filling resulted in more failure.

**Conclusions:**

The mixture of ZOE, iodoform and calcium hydroxide can be considered an effective root canal filling material in pulp involved primary teeth and had no adverse effect on tooth replacement.

**Trial registration:**

ChiCTR-TRC-14004938. Registered 13 July 2014.

## Background

According to the report of the third national oral health epidemiology investigation of China in 2005, 5-year-old children suffered from caries prevalence rate as high as 67% and the dmft index was 3.59 [1]. Endodontic treatment is considered the last option for keeping a primary tooth that has irreversibly affected pulp tissue due to caries in a child. The aim of pulpectomy is to preserve teeth in a symptom free state until they are replaced by their successor naturally during the transition from primary to permanent dentition, thus avoiding extraction. The adequate restoration of the involved teeth may preserve the arch length, reestablish the masticatory function and esthetics and prevent harmful tongue habits and speech alterations due to anterior teeth decay. The rationale includes the chemical and mechanical removal of irreversibly inflamed or necrotic radicular pulp tissue, followed by root canal filling with a material that can resorb at the same rate as the primary tooth and be eliminated rapidly if accidentally extruded through the apex [2].

Zinc oxide–eugenol (ZOE) was reported to more successfully manage those hyperemic primary pulp teeth than iodoform containing Ca(OH)_2_ pastes [3]. Nevertheless it has been reported to have a possibility to alter the path of eruption of the succedaneous tooth when it extrudes beyond the apex of the tooth because of forming a hard mass [4]. As far as resorption rate was concerned, ZOE often showed characteristic of slower rate than that of the primary teeth root resorption [5, 6].

Various clinical and radiographic success rates (65–100%) of iodoform have been reported [4, 7, 8]. However, Moskovitz et al. found that root resorption of primary molars is accelerated following pulpectomy with iodoform filling material in comparison with teeth without endodontic treatment [9].

The success rate with calcium hydroxide varied between 86.7 and 100% [10, 11]. Its main disadvantage is when used in primary teeth with hyperemic pulp, calcium hydroxide can come in contact with some vital pulp tissue remnants and can trigger the cascade of inflammatory root resorption [12].

Vitapex, which mainly contains calcium hydroxide and iodoform, were also discovered the early intraradicular resorption of the material, which means that the resorption rate is faster than that of the primary teeth root resorption [5, 13]. Though Nurko et al. found Vitapex was resorbed extraradicularly and intraradicularly without apparent ill effect, whether this affects the prognosis of pulpectomised teeth requires confirmation by further studies [14].

These root canal filling materials for primary teeth available at present do not fully meet the ideal requirements of primary teeth root canal treatment. The failure of root canal therapy in many primary teeth is due to inappropriate degradation of the material. The hypothesis of the study was adding the more resorbable materials (iodoform and calcium hydroxide) into ZOE would get favourable absorbability results of the mixture and improve the success rate of treatment. In this prospective study, a modified primary root canal filling (MPRCF), a mixture of ZOE, iodoform, and calcium hydroxide, was made and used in pulpectomies of primary molars in clinic. The aim of this paper is to evaluate the success rate of MPRCF, ZOE and Vitapex, which were the popular choose of dentists, as primary molar root canal filling materials and to compare the resorption rates.

## Methods

### Preparation of MPRCF

**Fig. 1:**
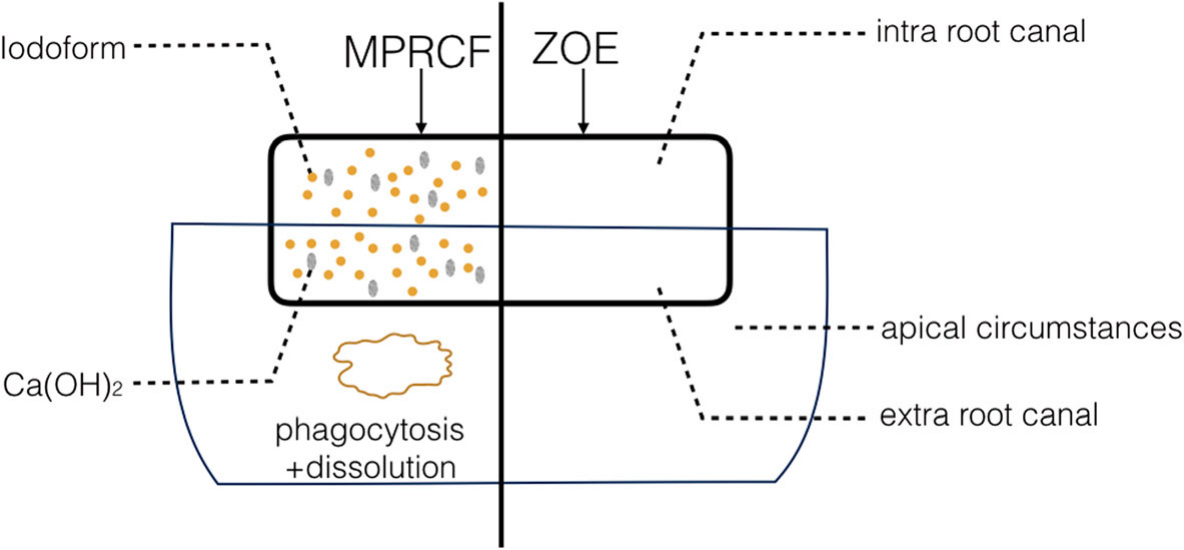
Schematic figure of MPRCF and ZOE. High solubility of calcium hydroxide, strength of chelation was lower and filling mass become porous when contacting with tissue fluid create the favourable resorb ability

In an in vitro study, the preparation of MPRCF was tested. The proportions of ZOE and iodoform were determined by the consistency of the paste and manufacturers guidance, approximately 1:1 in volume. Five different weight of calcium hydroxide, 0.01 g、0.02 g、0.03 g、0.04 g and 0.05 g, was added and the applicable setting time of mixture was recorded. Calcium hydroxide could accelerate the setting process and the more calcium hydroxide added, the shorter the setting time. The final amount of calcium hydroxide was determined in as low as 0.01 g. The eventual proportion of ZOE: iodoform: calcium hydroxide in the MPRCF was 0.28 g: 0.18 g: 0.01 g. The working time was 6 min and 50 s and setting time was 31 min, which was suitable for clinical use. The pH of MPRCF was 9.8. Zinc oxide, eugenol, iodoform and calcium hydroxide were provided by hospital preparation workroom. The schematic figure about the advantage of MPRCF compared with ZOE was showed in Fig. 1.

### Subjects

The present study was a double blind randomized controlled trail. The participants were collected in Department of Pediatric Dentistry, First Dental Center, Peking University School and Hospital of Stomatology. The name of trial registry was Chinese clinical trial registry and the registration number was ChiCTR-TRC-14004938. The Ethics Committee of Peking University School and Hospital of Stomatology approved this study. The parents/guardians of the participants signed individual informed consent forms containing information about the aim of the study and the treatment procedures.

Children between the ages of 4 and 9 were recruited and data concerning clinical and radiographic evaluation were collected in 6, 12 and 18 months or longer follow-up thereafter. The criteria for case selection were irreversible pulpitis, necrotic pulp or periodontitis as follow [15]:

1. Clinical characteristics, defined as spontaneous pain and the presence of a deep carious lesion with pulp exposure and bleeding that did not halt within five minutes following removal of the coronal pulp tissue. Gingival abscesses or fistula openings were absent or present. Abnormal mobility was requested.
2. Radiographic evaluation revealing that the molar had no internal resorption, with or without furcal or periapical radiolucencies and that physiological root resorption was less than 1/3.

Candidates were excluded if their molar could not be restored or if they had pathologic root resorption.

The sample size was calculated using the sample size formula in order to estimate a proportion. The clinical success rate in centre of ZOE was 85.7% reported by Gupta [13]. The desired accuracy of 10% and the significance level of 5% were considered. The sample size found was 47 children. Therefore, the sample of 51 children in each group used allowed a safety margin of 10% to account for possible losses to follow-up.

### Treatment, radiography, and follow-up

One dentist treated each molar involved in two visits. At the first appointment, the molar was isolated with a rubber dam following local anaesthesia. The pulp chamber was accessed after removal of all carious tooth structures. Pulpal debris was removed with barbed broaches. The working length was determined by superimposing an endodontic instrument over the preoperative radiograph and keeping it 1–2 mm short of the radiographic apex. Cleaning and shaping of the root canals was carried out using Mani k files (MANI Inc., Tochigi, Japan). The files were used sequentially in a pullback direction up to a maximum size of 35–40. Continuous irrigation with 2.5% sodium hypochlorite was carried out throughout the procedure.

Sterile paper points were used to dry the root canals. Calcium hydroxide (Multi-Cal, Pulpdent Corporation, Watertown, MA, USA) was injected into the root canal and a sterile cotton ball was placed in the pulp chamber and sealed with Cavit (3M ESPE, St. Paul, MN, USA) as temporary sealing material. At the second visit, which was typically two weeks later, the root canal was irrigated with 2.5% sodium hypochlorite and dried with sterile paper points. The prepared molars were allocated to ZOE, Vitapex (Neo Dental Chemical Products Co. Ltd., Tokyo, Japan), or MPRCF groups by random number table. The list was annexed in opaque covers and maintained the confidentiality of allocation until the time of obturation.

#### ZOE

Root canals were filled with ZOE paste (0.42 g zinc oxide, 0.14 g eugenol) mixed to medium consistency and delivered using lentulo spirals (MANI Inc.).

#### Vitapex

A syringe was inserted into the root canal near the apex and the material was extruded using moderate pressure. The syringe was then slowly withdrawn and the paste injected until it flowed back from the root canal into the pulp chamber.

#### MPRCF

We weighed 0.21 g zinc oxide (44.7%), 0.07 g eugenol (14.9%), 0.18 g iodoform (38.3%), and 0.01 g calcium hydroxide (2.1%) using an electronic scale, then combined them in sequence to medium consistency, packed it into the root canal using lentulo spirals.

Radiographs were obtained to determine whether the root canals were completely filled. The root canal fillings were categorised with regard to length in relation to the radiographic apex: underfilled (longer than 2 mm), adequate (0–2 mm), or overfilled (over apex). A tooth was categorized as overfilled even if other roots were filled short or adequate [16]. The pulp chambers of all molars were restored with GIC (Lime-Lite Light Cure Cavity Liner, Pulpdent Corporation) and cavities were restored with composite resin (Filtek Z250 Universal Restorative, 3M ESPE) or 3M stainless steel crown.

Molars were evaluated clinically and radiographically at 6, 12 and 18 months later by two investigators blinded to the type of filling material used for each molar. The patients and their guardians also didn’t know which group they allocated in. The teeth would be refilled if any recurrent caries present to ensure no leakage. The intra-examiner reliability of the first and second co-investigator was calculated by Cohen’s kappa statistic (0.85 and 0.95, respectively). The evaluations of the two co-investigators were calibrated and standardised to determine inter-examiner reliability by independently analysing the radiographs of 20 primary molars. Cohen’s kappa statistic indicated excellent reproducibility between the two co-investigators with a measurement agreement of 0.85.

### Clinical and radiographic success criteria

The criteria for clinical success were that patients were completely free of clinical signs and symptoms including pain, gingival abscesses, fistula openings, and abnormal mobility [17, 18].

The criteria for radiographic success were no pathologic external root resorption and no radiographic lesions [7, 19]. The radiographic examination also included resorption of excess extraradicularly extruded materials and filling material in the root canal, and direction of the successor permanent molars. The overfilled material was described as non-resorbed, partly resorbed, or completely resorbed. The state of filling material in the root canal were divided into five levels: both root and filling no change; root no change but filling resorbed; root began resorption, filling resorbed at faster rate; root began resorption, filling resorbed at same rate (the distance from the apex in the radiograph to the bottom of the filling being less than 1 mm); root began resorption, filling resorbed at slower rate [20].

### Statistical analysis

Statistical analysis was performed using SPSS 17.0. The Chi-square Test was used to compare the distribution and success rate difference between the three groups. Relationship of faster resorption of materials in the root canals and radiographic success in Vitapex group at 18 months was determined with Fisher’s exact test. Comparison of resorption of overfilled material between ZOE and MPRCF group used Mann–Whitney U test. A *P*-value <0.05 was considered statistically significant.

## Results

**Fig. 2:**
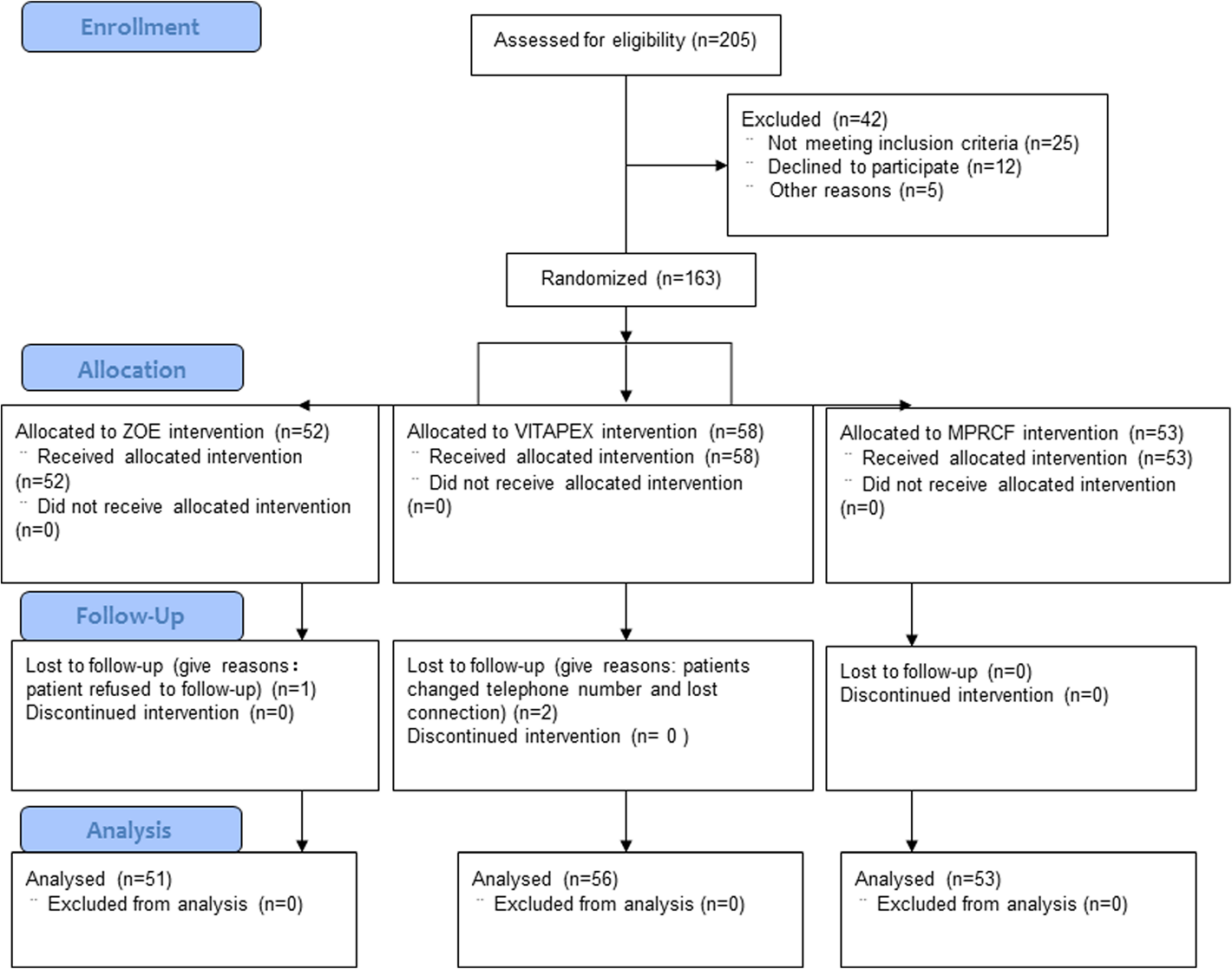
Flowchart of the present clinical study

The final sample of the clinical trial consisted of 155 children (average age 5.88 ± 1.27 years), 160 molars in total. The loss of follow up rate was 1.84%. The participant CONSORT flow diagram was shown in Fig. 2. The distribution of the 160 primary molars at baseline is equilibrium in age, gender, first or second molars and maxillary or mandibular between groups.

Clinical and radiographic success rates of ZOE, Vitapex, and MPRCF root canal treatment at 6,12 and 18 months were showed in Table 1. No molar in the ZOE and MPRCF groups failed the 6- and 12-month clinical and radiographic evaluations. At 18-month evaluation six molars in ZOE group and four molars in MPRCF groups showed radiologically failed compared with 26 molars failure (46.4%) in Vitapex group. There was a significant difference in clinical and radiographic success rate between the ZOE and Vitapex groups (*P* = 0.01) and between the MPRCF and Vitapex groups (*P* = 0.01) at 12 months and 18 months. There were no significant differences between MPRCF and ZOE group at any time-point.

**Table 1:** Clinical and radiographic success rates of ZOE, Vitapex, and MPRCF root canal treatment at 6,12 and 18 months

	Clinical success						Radiographic success					
	6 months		12 months		18 months		6 months		12 months		18 months	
	N	%	N	%	N	%	N	%	N	%	N	%
ZOE	51	100	51	100^a^	47	92.2^a^	51	100	51	100^a^	45	88.2^a^
Vitapex	56	100	45	80.4^ab^	40	71.4^ab^	53	94.5	34	60.7^ab^	30	53.6^ab^
MPRCF	53	100	53	100^b^	51	96.2^b^	53	100	53	100^b^	49	92.5^b^

^a^mean the success rate were significantlly different between ZOE group and Vitapex group

^b^mean the success rate were significantlly different between Vitapex group and MPRCF group

**Fig. 3:**
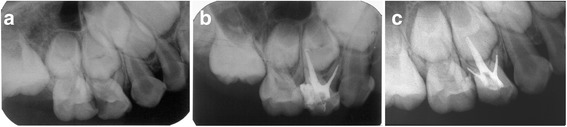
Eighteen months follow-up of MPRCF. **a** Pre–operative radiograph of 54 considered for root canal treatment with MPRCF. **b** Radiograph taken immediately postoperatively showing adequate filled. **c** Radiograph 18 months postoperatively showing the fillings were resorbed at the similar rate with palatal root

**Fig. 4:**
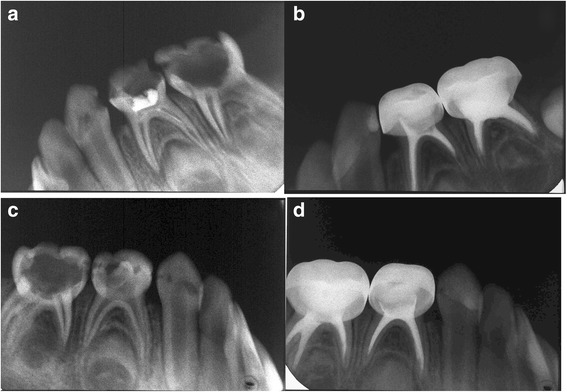
Eighteen months follow-up of MPRCF. **a, c** Pre–operative Radiograph of *right* and *left* mandibular first and second primary molar considered for root canal treatment with MPRCF. **b**, **d** Radiograph 18 months postoperatively showing the MPRCF fillings were stable and intact

**Fig. 5:**
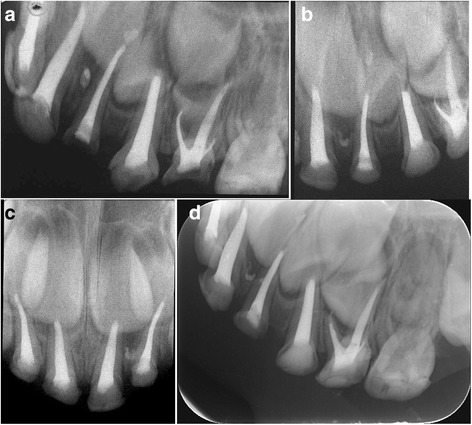
Twenty-four months follow-up of MPRCF. **a** Radiograph of 52–64 treated with MPRCF taken 6 months postoperatively. **b**, **c** Radiograph taken 18 months postoperatively showing partly resorption of overfilled materials in 62. **d** Radiograph taken 24 months postoperatively showing intact filling

**Fig. 6:**
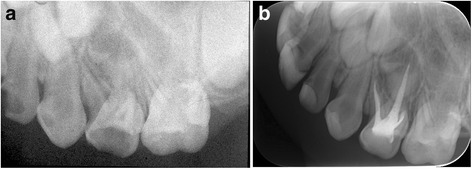
Eighteen months follow-up of MPRCF. **a** Pre–operative radiograph of maxillary first left primary molar of a 3-year-old girl considered for root canal treatment with MPRCF **b** Radiograph taken 18 months postoperatively showing no resorption of materials in all roots canal

**Fig. 7:**
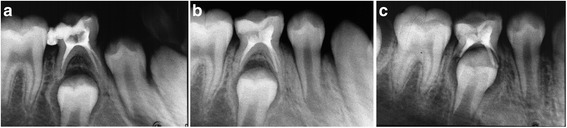
Twelve months follow-up of ZOE. **a** Radiograph of 84 treated with ZOE taken immediately postoperatively showing adequate filled. **b** Radiograph taken 6 months postoperatively showing slower resorption rate of filling material than roots. **c** Radiograph taken 12 months postoperatively showing remaining of filling material

More teeth filled with MPRCT showed the same resorption rate between the root filling material and roots. In the MPRCF group, when roots had begun physiological resorption, the filling material was resorbed at the same rate in 87.1% molars at 18 months (Fig. 3). During the roots were in stable stage, the MPRCF filling inner the roots kept intact synchronously (Figs. 4, 5 and 6). ZOE was resorbed slowly than roots in 39.2% teeth at 18 months (Fig. 7). The Chi-square test showed that at 18 months there was a significant difference of the proportions of resorption at same rate between the MPRCF and ZOE (*P* = 0.001) and the MPRCF and Vitapex groups (*P* = 0.01) (Table 2).

**Table 2:** Relationship of resorption rates of materials in the root canals and roots

	ZOE		Vitapex		MPRCF	
	N	%	N	%	N	%
6 months						
A	48	94.1	23	41.1	32	68.2
B	0	0	27	48.2	0	0
C	0	0	2	3.6	0	0
D	2	3.9 ^a^	4	7.1 ^b^	21	31.8 ^ab^
E	1	2.0	0	0	0	0
12 months						
A	32	62.7	10	17.9	26	49.1
B	0	0	14	25	0	0
C	0	0	28	50	3	5.7
D	3	5.9^a^	4	7.1^b^	24	45.2^ab^
E	16	31.4	0	0	0	0
18 months						
A	25	49.0	7	12.5	20	37.7
B	0	0	16	28.6	2	3.6
C	0	0	31	55.4	4	7.5
D	6	11.8^a^	2	3.5^b^	27	50.9^ab^
E	20	39.2	0	0	0	0
Total	51	100	56	100	53	100

A: both no changes, B: root no change but filling resorbed, C: root began resorption, filling resorbed at faster rate, D: root began resorption, filling resorbed at same rate, E: root began resorption, filling resorbed at slower rate

^a^mean the proportions of resorption at same rate were significantlly different between ZOE group and MPRCF group

^b^mean the proportions of resorption at same rate were significantlly different between Vitapex group and MPRCF group

At 18 months, the proportion of molars in which Vitapex was resorbed faster than the root was 84% and 26 molars of them failed in radiographic evaluation. Fisher’s exact test showed the situation of Vitapex was resorbted faster than root resulted in more failure pulpectomy than non-resorbed condition, at least in radiographic evaluation (Table 3).

**Table 3:** Relationship of faster resorption of materials in the root canals and radiographic success in Vitapex group at 18 months

	Faster filling resorption		
	+	_	*P*
Success	21	9	
Fail	26	0	0.0001

The resorption of overfilled materials wsa showed in Table 4. Extruded Vitapex was resorbed completely at 6-month follow-up. Two-thirds extruded ZOE was not resorbed at 12 months and about one-thirds at 18 months. Extruded MPRCF was completely resorbed in 72.7% of cases at 12 months and 100% at 18 months. There were significant difference of the resorption of overfilled material between ZOE and MPRCF group.

**Table 4:** Comparison of resorption of overfilled material between ZOE and MPRCF group

	ZOE		MPRCF		*P*
	N	%	N	%	
6 months					
Non resorb	15	71.4	0	0	
Partly resorb	5	23.8	12	54.5	
Completely resorb	1	4.8	10	45.5	0.000
12 months					
Non resorb	14	66.7	0	0	
Partly resorb	4	19.0	6	27.3	
Completely resorb	3	14.3	16	72.7	0.000
18 months					
Non resorb	8	66.7	0	0	
Partly resorb	8	19.0	0	0	
Completely resorb	5	14.3	22	100	0.000
Total	21	100	22	100	

## Discussion

Clinical studies have shown the success rate of ZOE paste used alone to range from 54 to 100% [6, 19–21] and there is no difference between the success rates of ZOE, calcium hydroxide, or iodoform paste [19, 21]. The bacterial leakage test model of Sisodia R et al. indicated that Zinc Oxide Eugenol showed no bacterial leakage and better resistance to bacterial leakage than Apexit plus (a calcium hydroxide based root canal sealer paste) [22]. ZOE showed better inhibitory activity against most of the organisms isolated than Vitapex, Calcium hydroxide and Metapex, which proved by Harini PM et al. [23]. These characteristics may be the cause of good clinical manifestations of ZOE, and was also the reason why the present study chooses the ZOE as the main component of the mixed paste.

However, particles of extruded material remained evident after 18 months in most cases in the ZOE group, which was consistent with the findings of previous studies [4–6, 24]. Several researchers found that ZOE extruded extraradicularly was resorbed slowly and might need several months or even years [5, 25, 26]. Allen explained the reason might be the ability of ZOE to resist phagocytising macrophages [27]. With respect to the negative effect of extruded ZOE, Ozalp et al. and other investigators observed that ZOE caused the permanent tooth germ to erupt abnormally [6, 26].

The relatively high number of overfilled molars for the ZOE (41.2%) and MPRCF (41.5%) groups was caused by the use of lentulo spirals and the consistency of pastes. There is no apical constriction of root in primary teeth and measuring the working length in primary teeth is relatively difficult also contributed to the extrusion of material.

In this study, patients with Vitapex that extruded beyond the apex all exhibited complete resorption within six months, which consisted with other researchers’ findings [14, 28, 29]. Vitapex had been used as root filling material in primary tooth pulpectomies for many years until rescently researchers have observed that Vitapex resorption was faster than root canal in follow-up period [6, 21, 29]. Ramar reported 56.6% of Vitapex treated teeth showed material resorption ahead of the roots in 9 months [30]. In the present study, when Vitapex was resorbed faster than roots, 55% teeth failed in radiographic evaluation and 61% the radiographic-failed cases exhibited clinical signs and symptoms. Our study confirmed that the excessive resorption rate of root canal filling affected both clinical and radiographic success rate. Though Nurko et al. found Vitapex was resorbed faster than roots without apparent ill effect [14], the authors mentioned a longer follow-up is recommended to evaluate if there is any effect on the permanent succedaneous tooth. Actually the early resorption of Vitapex may form a narrow channel for bacterial growth and cause reinfection in the root canal [5]. Nakornchai and Banditsing also revealed the clinical success rate of Vitapex could as high as 96%, but the 6- and 12-month radiographic success rates were 80 and 56% respectively; their findings were similar to ours [16].

Root canal filling material of primary teeth should be resorbed at an identical rate, or as similarly as possible, to that of physiological root resorption. This study used a modified paste comprising a mixture of ZOE, iodoform, and calcium hydroxide as root canal filling material in primary molars. Our results indicated that the modified paste with a success rate of 92.5% is a much better material compared with Vitapex and had better absorbability compared with ZOE alone. The possible reason was that the mixture does not set into a hard mass. The potential mechanism lied in two aspects. Firstly, the essence of formation of ZOE is the reaction of eugenol and bivalent zinc ions to form insoluble chelation, wrapping remanent zinc oxide in it and forming a solid mass. Because calcium ion dissolves more easily than zinc ions, adding calcium hydroxide forms divalent metal chelate salt containing mainly eugenol calcium. Owing to the high solubility of calcium hydroxide, the reaction time is shorter, but strength of chelation was slightly low, thus degrade more quickly. Secondly, iodoform dissolves easily upon contact with solutions and tissue fluid, changing the structure of the filling mass to a porous and loose state that might be resorbed more easily [14].

Interestingly, Endoflas, produced in South America, also comprising of triiodomethane, zinc oxide eugenol, calcium hydroxide, has been reported having the resorption limited to the excess material [17] and resorbs at the same pace as the physiological resorption of root [15]. The antimicrobial efficacy in-vitro study showed Endoflas had good antimicrobial potential against eight microbial strains including E. faecalis compared to other primary root canal filling materials [31, 32]. Specifically, MPRCF had the advantage of resorption that was limited to the material extruded extraradicularly without intraradicular early resorption. As the property of resorption, the MPRCF fulfilled the basic requirement of an ideal root canal filling material for primary molars more compared with ZOE. However, further in-depth study is need to complete the in-vitro antibacterial effects of this combination and understanding the degradation mechanism of this mixed root filling material and longitudinal study involving a larger sample size is necessary to evaluate the success and resorpsion rate until the teeth’ eventual exfoliation.

## Conclusion

Endodontic treatment using a mixture of ZOE, iodoform, and calcium hydroxide in primary teeth has shown better clinical and radiographic success than Vitapex at 12 and 18 months and had similar success rate with ZOE. The MPRCF can be considered an effective root canal filling material in primary teeth due to its better resorbable characteristics.

## Abbreviations

MPRCF: Mixed primary root canal filling
ZOE: Zinc oxide–eugenol
